# Immune Checkpoint Inhibitors in pMMR Metastatic Colorectal Cancer: A Tough Challenge

**DOI:** 10.3390/cancers12082317

**Published:** 2020-08-17

**Authors:** Federica Marmorino, Alessandra Boccaccino, Marco Maria Germani, Alfredo Falcone, Chiara Cremolini

**Affiliations:** 1 Department of Translational Research and New Technologies in Medicine and Surgery, University of Pisa, via Risorgimento 36, 56126 Pisa, Italy; f.marmorino@studenti.unipi.it (F.M.); a.boccaccino@studenti.unipi.it (A.B.); m.germani@studenti.unipi.it (M.M.G.); alfredo.falcone@med.unipi.it (A.F.); 2 Unit of Medical Oncology, Azienda Ospedaliera Universitaria Pisana, Via Roma 67, 56126 Pisa, Italy

**Keywords:** metastatic colorectal cancer, immune checkpoint inhibitors, microsatellite stable, proficient DNA mismatch repair

## Abstract

The introduction of checkpoint inhibitors provided remarkable achievements in several solid tumors but only 5% of metastatic colorectal cancer (mCRC) patients, i.e., those with bearing microsatellite instable (MSI-high)/deficient DNA mismatch repair (dMMR) tumors, benefit from this approach. The favorable effect of immunotherapy in these patients has been postulated to be due to an increase in neoantigens due to their higher somatic mutational load, also associated with an abundant infiltration of immune cells in tumor microenvironment (TME). While in patients with dMMR tumors checkpoint inhibitors allow achieving durable response with dramatic survival improvement, current results in patients with microsatellite stable (MSS or MSI-low)/proficient DNA mismatch repair (pMMR) tumors are disappointing. These tumors show low mutational load and absence of “immune-competent” TME, and are intrinsically resistant to immune checkpoint inhibitors. Modifying the interplay among cancer cells, TME and host immune system is the aim of multiple lines of research in order to enhance the immunogenicity of pMMR mCRC, and exploit immunotherapy also in this field. Here, we focus on the rationale behind ongoing clinical trials aiming at extending the efficacy of immunotherapy beyond the MSI-high/dMMR subgroup with particular regard to academic no-profit studies.

## 1. Introduction

The introduction of immunotherapy with Programmed Death 1, Programmed Death-Ligand 1 (PD-1 or PD-L1) or Cytotoxic T-Lymphocyte Antigen 4 (CTLA-4) immune checkpoint inhibitors has deeply transformed the management of many cancers, leading to remarkable improvements in terms of survival and radiological response for some patients [1].

In metastatic colorectal cancer (mCRC) long-lasting responses from immune checkpoint inhibitors are restricted to a small group, approximately 5%, of patients, i.e., those harboring microsatellite instability (MSI-H)/deficient DNA mismatch repair (dMMR) tumors [2,3].

In refractory dMMR mCRC subgroup, both low dose ipilimumab (anti CTLA-4 agent) plus nivolumab (anti PD-1) combination or nivolumab alone have been granted FDA approval [4,5].

Recent results of the phase III KEYNOTE 177 study are likely to change the treatment algorithm of the first line therapy for untreated dMMR mCRC patients, since in the first randomized comparison, pembrolizumab was demonstrated superior to the first-line standard of care with a doubling in terms of median Progression-Free Survival (PFS) [16.5 vs. 8.2 months, hazard ratio (HR) = 0.60; *p* = 0.0002] [6]. Based on these results, on 29 June 2020 pembrolizumab (anti PD-1) has been approved by FDA for the first-line treatment of patients with unresectable or metastatic dMMR CRC [7].

The success of immune checkpoint inhibitors in dMMR tumors has been linked to their hypermutated phenotype characterized by high mutational load as a result of multiple DNA replication errors and to the consequent inflammatory tumor microenvironment (TME) consisting of a great amount of tumor infiltrating lymphocytes (TILs), especially memory cells and cytotoxic T lymphocytes (CTLs), effectors of the anti-tumor immune response [8,9,10,11,12].

Pembrolizumab has recently received a new agnostic indication for the treatment of refractory patients with unresectable or metastatic solid tumors with high tumor mutational burden (TMB-H) [≥10 mutations per megabase (mut/Mb)] [13].

This accelerated approval is based on findings from an analysis of 10 cohorts of patients with previously treated metastatic solid tumors with TMB-H enrolled in KEYNOTE-158 trial, which explores the use of pembrolizumab until unacceptable toxicity or disease progression. Of note no patients bearing colorectal cancer were included. Among the 790 patients, 102 (13%) had tumors identified as TMB-H, defined as TMB ≥ 10 mut/Mb. The response rate of TMB-H patients was 29% (vs. 6% in patients with a TMB < 10) with complete responses in 4% of patients [14].

The relationship between TMB and response to immunotherapy is explained by the generation of neoepitopes typical of tumors with many mutations that become a target for antigen presentation and powered CTLs activity [15]. 

Conversely, checkpoint inhibitors failed to provide benefit to the majority of mCRC defined as pMMR (about 95% of mCRC) that show a considerably lower TMB and an immune excluded or immune desert TME with absent or inactive CTLs and a reduced expression of checkpoint proteins [9,16,17].

Hypothesizing better results from the synergistic activity of more checkpoint inhibitors with different mechanisms of action, the combination of the anti PDL-1 agent durvalumab with the CTLA-4 inhibitor tremelimumab was evaluated in chemorefractory pMMR mCRC patients. This phase II trial reported a small hint of activity of the checkpoint inhibitors combination compared with best supportive care, but with modest clinical benefit, confirming that checkpoint inhibitors alone or in association have no chance to overcome the intrinsic resistance of pMMR mCRC. Indeed, even after dissecting patients according to their TMB (≥ vs. <20 mut/Mb), no interaction between TMB and treatment received was detected [18,19]. 

Understanding the wide variety of mechanisms that primarily inhibit the activation of immune system in response to checkpoint inhibitors in pMMR mCRC is the keystone for biologically sound combination therapies.

The development of an effective immune response against tumor cells requires multiple steps from the activation of the effector immune cells to their recruitment within the TME. Novel strategies should prompt the immune cycle at various levels, either by transforming TME into an immune responsive phenotype with efficacious immune infiltrate, and/or by increasing the mutational load and neoantigens generation, or by influencing the interferon-γ (IFN-γ) signature with inhibition of immunosuppressive ligands expression.

To this end, cytotoxic agents, target therapies and/or radiotherapy might affect the interplay among cancer cells, tumor infiltrating stroma and host immune system [9,20].

**Figure 1:**
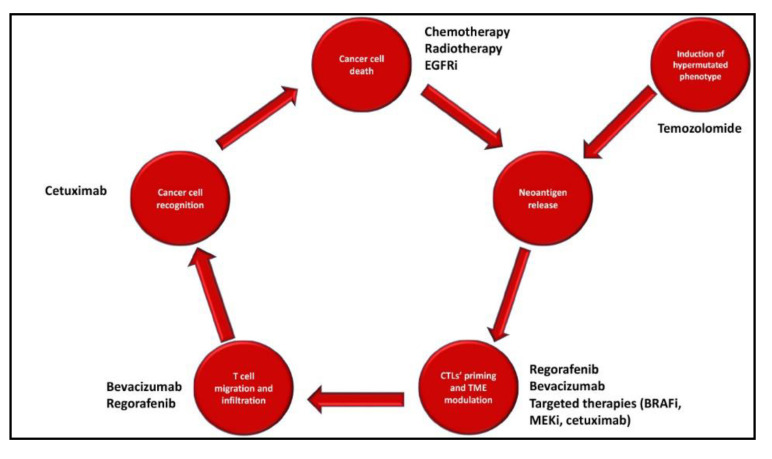
Approaches to enhance immunogenicity of proficient mismatch repair (pMMR) colorectal cancer. CTL: cytotoxic Lymphocytes; BRAFi: BRAF inhibitors; EGFRi: EGFR inhibitors; MEKi: MEK inhibitors; TME: Tumor Microenvironment.

Although several experiences reported disappointing results, other promising approaches require further evaluation in order to build an effective antitumoral immune response in pMMR mCRC tumors (Figure 1). Here, we discuss current knowledge in the field of combination strategies with checkpoint inhibitors in pMMR mCRC focusing on approaches in advanced phases of clinical research (Table 1 and Table 2).

## 2. Anti-VEGF and Chemotherapy + Immune Checkpoint Inhibitors

One potential mechanism to convert resistance in pMMR mCRC is to recruit CTLs from the blood among TILs and to favor the recognition of malignant cells by CTLs in TME, thus modifying tumor immune contexture and favoring checkpoint inhibitors effect [58,59,60].

In preclinical experiences, chemotherapy induces immunogenic cell death and the subsequent release of neoantigens that are recognized by dendritic cells able to present them to CTLs and, therefore, to activate immune response against cancer cells [61,62,63,64].

Among cytotoxic agents used in mCRC, 5-fluorouracil (5FU) selectively determines apoptosis of myeloid-derived suppressor cells (MDSCs) favoring CTLs activity and it induces tumor cell death that renders tumor cells recognizable by the immune system [65,66]. Oxaliplatin also induces immunogenic cell death promoting presentation of cancer-specific antigens and shifting the balance between effector/regulatory cells in favor of effector cells [67,68,69].

Moving from this biological rationale, FOLFOX regimen in addition to pembrolizumab was evaluated in a phase II study achieving an overall response rate (ORR) of 53% with a disease control rate (DCR) of 100% at 8 weeks in patients with untreated mCRC including 3 with dMMR, 22 pMMR, and 5 with no available data (NCT02375672) [21].

Nowadays, the anti-Vascular Endothelial Growth Factor A (VEGF-A) agent bevacizumab, in combination with chemotherapy, plays a crucial role in the treatment of mCRC patients. VEGF can directly trigger T regulatory cells (T-reg) proliferation, implementing MDSCs infiltration and it may promote CTLs exhaustion by up-regulating suppressive immune checkpoint molecules, such as PDL-1 [70,71,72].

Indeed bevacizumab, by inhibiting VEGF-VEGFR pathway, stimulates the maturation of dendritic cells (DCs) and reduces the expansion of T-reg lymphocytes and MDSCs thus contributing to immune effector cells activation. Moreover, the vasculature normalization allows an increasing in CTLs tumor infiltration.

Taken together, these data suggest that chemotherapy plus bevacizumab could be a tool to shift cold into hot (inflamed) tumors [73,74].

Nevertheless, up today results of trials assessing this therapeutic strategy are disappointing. In the MODUL trial, after an induction treatment with FOLFOX/bevacizumab, the addition of the anti-PD-L1 agent atezolizumab to maintenance therapy with 5FU/bevacizumab did not improve PFS and overall survival (OS) in BRAF wild type pMMR mCRC patients [22].

Consistently, unsatisfactory results were reported with the addition of atezolizumab to the oral fluoropyrimidine capecitabine and bevacizumab in the chemorefractory setting, providing a statistically significant modest improvement in PFS in the BACCI trial (4.4 vs. 3.3 months, HR = 0.72; *p* = 0.051) [23].

Moving to the earlier lines of therapy, the combination of atezolizumab with bevacizumab and a doublet oxaliplatin-based chemotherapy (FOLFOX) did not show unexpected adverse events or exacerbation of FOLFOX or bevacizumab-related toxicities in a phase Ib trial enrolling untreated mCRC patients, though the absence of a control arm does not allow drawing any definitive conclusion on safety results [24]. 

Based on the phase III TRIBE study demonstrating the superiority of the triplet FOLFOXIRI plus bevacizumab compared with the doublet FOLFIRI plus bevacizumab in previously untreated mCRC patients, the intensified treatment plus bevacizumab is recognized as a standard option as first-line therapy in selected mCRC patients [75].

The intensification of the chemotherapy backbone can boost the release of novel neoantigens and T-cell infiltration, thus increasing the likelihood of response to immunotherapy compared to doublet regimen.

In a retrospective analysis of immunological parameters assessed on CRC liver metastases resected after pre-operative intensified chemotherapy plus bevacizumab or cetuximab, TME-driven immune response was associated with better survival regardless of other well-known prognostic factors. The intensified chemotherapy regimen may contribute to the activation of the immune phenotype, thus supporting the combination of immunotherapy with FOLFOXIRI in first-line therapy in order to establish a favorable TME for immunotherapy action [76].

Drawing from this rationale, the ATEZOTRIBE study is being conducted and has recently completed the planned accrual. This academic study is a multicenter (around 30 Italian sites) randomized comparative trial in which mCRC patients are randomized to receive FOLFOXIRI plus bevacizumab (standard arm) or FOLFOXIRI plus bevacizumab with atezolizumab (experimental arm) as first-line treatment, irrespective of tumor microsatellite status. Patients are treated with an induction phase of 8 cycles followed by maintenance with 5FU plus bevacizumab in the standard arm or 5FU plus bevacizumab and atezolizumab in the experimental arm until progression. PFS is the primary endpoint [25]. In the TRIBE2 study upfront FOLFOXIRI/bevacizumab followed by the reintroduction of the same agents after disease progression demonstrated superior PFS2 (defined as the time from randomization until the evidence of disease progression on any treatment given after first progression) and OS compared with FOLFOX/bevacizumab followed by FOLFIRI/bevacizumab after progression [77]. Based on these results, the reintroduction of the upfront treatment after disease progression is planned in the ATEZOTRIBE trial [25]. 

According to the same rationale, NIVACOR is an Italian trial designed with the purpose to explore the combination of FOLFOXIRI plus bevacizumab with nivolumab in patients with RAS/BRAF mutant mCRC, regardless of microsatellite status (NCT04072198) [32]. In this multicenter study, RAS/BRAF mutant mCRC patients are currently recruited to receive up to 8 cycles of FOLFOXIRI plus bevacizumab plus nivolumab as first-line therapy followed by maintenance with bevacizumab plus nivolumab. The primary endpoint is ORR and the study is designed with the hypothesis to improve the ORR from 66% (expected according to literature data with FOLFOXIRI plus bevacizumab) to 80% [32].

## 3. Anti-EGFR ± Chemotherapy + Immune Checkpoint Inhibitors

Targeting the Epidermal Growth Factor Receptor (EGFR) with the monoclonal antibodies cetuximab or panitumumab administered alone or in combination with chemotherapy is an efficacious strategy in the treatment of RAS wild type mCRC [78,79].

Besides its targeted effect, cetuximab, a chimeric IgG1 antibody, interacts with effector immune cells through a Fc region dependent mechanism leading to the formation of immune complexes and inducing antibody-dependent cellular cytotoxicity (ADCC), a major host defense mechanism against tumors. Additionally, cetuximab can induce the activation of natural killer (NK) cells with subsequent increase of CTLs/T-reg cells ratio among TILs [80,81,82]. 

The other anti-EGFR agent, the fully human IgG2 antibody panitumumab, does not show the same ability in mobilizing innate and adaptive immune cells against tumor cells [83].

The phase II AVETUX study demonstrated high response rate (ORR: 79.5%) with the combination of FOLFOX and cetuximab with the anti PDL-1 agent avelumab as first-line therapy for RAS/BRAF wild type mCRC patients independently of the microsatellite status (5% of included tumors were dMMR), though missing its primary endpoint (increase in progression-free rate at 12-month from 40% to 57%), having only 40% patients not experienced disease progression at the established timepoint [26]. As already shown in other studies that investigated anti-EGFR regimens, these activity results did not translate into similar gains in delaying tumor progression, challenging the role of PFS as surrogate endpoint for OS for this treatment. In addition, the suboptimal performance of RECIST criteria in the definition of the response from immunotherapy is well known [26]. 

The central tissue review detected low frequent RAS mutations in three enrolled patients and in two of these patients partial response was reported, underlining the activity of this combination also in a population potentially resistant to anti-EGFR agents and with poorly inflamed TME [26]. However, the clinical relevance of low-frequency RAS mutations on the response to anti-EGFRs remains undefined, and the co-administration of FOLFOX does not allow excluding the effect of the chemotherapy backbone, independently from a possible synergistic effect of avelumab and cetuximab, as a potential confounding factor [84,85]. Future translational research should explore whether the immunomodulatory properties of cetuximab are independent of its on-target activity (EGFR), thus investigating its off-target immunologic effect in RAS/BRAF mutated patients.

The modified schedule of the triplet FOLFOXIRI in combination with an anti-EGFR agent (cetuximab or panitumumab) showed interesting activity with a manageable safety profile in RAS/BRAF wild type mCRC patients. In the randomized phase II VOLFI trial, FOLFOXIRI plus panitumumab provided a clear advantage in ORR and secondary resection rate compared with FOLFOXIRI alone (87.3 vs. 60.6%, *p* = 0.004 and 33.3 vs. 12.1%, *p* = 0.02, respectively) [86].

Based on these results, the AVETRIC study has been launched in order to provide a preliminary evaluation of a new therapeutic strategy combining the intensified chemotherapy regimen plus the anti-EGFR cetuximab and the anti-PD-L1 avelumab [33]. 

In this ongoing phase II single-arm study, RAS wild-type mCRC patients receive up to 12 cycles of modified FOLFOXIRI plus cetuximab and avelumab as first-line therapy, followed by maintenance with 5-FU plus cetuximab and avelumab, regardless of microsatellite status. The primary endpoint is PFS [33].

The potential synergic effect of an anti-EGFR plus a checkpoint inhibitor is currently being tested also in later lines of therapy. Among pMMR and anti-EGFR-naïve patients, a phase II study is exploring the combination of nivolumab and ipilimumab with panitumumab (NCT03442569) [87]. 

Another field of improvement of the efficacy of anti-EGFR agents in the continuum of care of mCRC is the re-treatment in advanced lines of patients already exposed with benefit to a first-line anti-EGFR-containing regimen [88,89]. Phase II studies evidenced the activity of the rechallenge with cetuximab in patients with RAS/BRAF wild-type mCRC who achieved response to the upfront chemotherapy plus anti-EGFR, then became resistant to the treatment, and received an anti-EGFR-free therapy after progression [88,89]. 

Drawing from this evidence, the phase II single-arm CAVE Colon trial (Eudract Number 2017-004392-32) is exploring whether the addition of avelumab may potentiate the clinical efficacy of rechallenge with cetuximab in the third-line setting. The primary endpoint is OS and patients are not selected for microsatellite status [27].

## 4. Temozolomide + Immune Checkpoint Inhibitors

The peculiar genomic landscape of dMMR tumors and the higher expression of neoantigens serving as immunogenic epitopes able to trigger immune response is one of the explanations of the efficacy of immune checkpoint inhibitors in these tumors unlike pMMR ones [60].

Some anti-tumor agents, such as temozolomide (TMZ), may be able to induce a higher rate of somatic mutations in tumor cells resulting in an inflamed TME, thus generating a microsatellite-instable-phenotype in sensitive pMMR mCRC [90]. 

TMZ is an oral alkylating agent that acts by methylating DNA strands at the O6 position of guanine. This methylation damages DNA, inducing inhibition of replication and apoptosis. The O6-methylguanine methyltransferase (MGMT) enzyme coded by the MGMT gene reduces the therapeutic efficacy of TMZ [91].

The epigenetic silencing of MGMT, mediated by the methylation of its promoter region, is involved in a diminished DNA-repair of O6-alkylguanine adducts, and it is able to enhance the sensitivity of cancer cells to alkylating agents [92,93]. 

TMZ is indicated for the treatment of patients with glioblastoma, for which MGMT methylation has been strongly associated with better response from TMZ [94,95,96,97]. MGMT methylation is also detected in approximately 30–40% of CRC and it is strongly associated with RAS mutations [92].

Although phase II trials showed that TMZ is an active option in some pretreated patients with mCRC, it is not yet recognized as a standard option in current guidelines due to the lack of a formal comparison, the use of different drug schedules and heterogeneous techniques of detection of MGMT methylation and cut-off levels to define hypermethylation [98,99,100,101,102,103].

Moreover, not all patients with MGMT hypermethylated tumors achieved clinical benefit from TMZ, highlighting the need to optimize the evaluation of MGMT methylation as a predictive marker of response.

A retrospective analysis identified the contemporary presence of MGMT hypermethylation and MGMT low/absent expression as the best selection criterion to identify patients more likely to benefit from alkylating agents [97]. 

Recent preclinical data suggest that TMZ is able to induce somatic mutations in mismatch repair genes in solid tumors [104,105]. In CRC cells initially responsive to TMZ, at the time of acquired resistance a hypermutated phenotype is evident together with a high load of neoantigens translating into a dMMR-like phenotype [90]. 

Other studies indicated that alkylating agents may play a role in immunogenic stimulation resulting in a selective depletion of T-reg lymphocytes and in the activation of CTLs [106].

The promising activation of an effective immune surveillance by TMZ is the rationale behind two currently ongoing trials that investigate the unexplored strategy of combining TMZ and immune checkpoints inhibitors in pMMR mCRC patients.

ARETHUSA (NCT03519412) is a 2-cohort not randomized phase II study in which refractory mCRC patients with dMMR tumors receive pembrolizumab until progression and patients with pMMR RAS mutated, MGMT IHC-negative/promoter hypermethylation positive mCRC are treated with TMZ until progression. 

Tumor biopsy is performed at the time of disease progression in the pMMR cohort to determine TMB, and patients receive pembrolizumab if TMB is >20 mutations/Mb [34]. 

The working hypothesis underlying ARETHUSA is that the acquired resistance to TMZ is responsible for the onset of a hypermutated phenotype predictive of response to pembrolizumab. The primary endpoint is ORR in pMMR treated with pembrolizumab, while dMMR cohort will be used for indirect comparison [34]. 

Another study in this setting is MAYA (NCT03832621), a phase II single-arm trial designed to evaluate the activity of the combination of nivolumab, ipilimumab and TMZ in patients with pMMR, MGMT-silenced mCRC who are not progressed after two cycles of single-agent TMZ. Patients are eligible independently of RAS mutational status [35].

Based on the ability of TMZ to induce microsatellite-instable-phenotype-like status, both directly, by favoring CTLs activation, and indirectly, by increasing the TMB, MAYA aims at investigating the potential sensitization to immunotherapy in patients achieving disease control from an initial TMZ treatment [35]. 

Differently from ARETHUSA, in MAYA trial the immunotherapy is administered in combination with TMZ in patients with no disease progression after two cycles of TMZ rather than after the evidence of acquired resistance [35].

Indeed, the primary endpoint is the 8-month PFS rate with the combination of TMZ, nivolumab and ipilimumab in patients achieving disease control [complete or partial response or stability of disease (CR, PR, SD)] after 2 months of treatment with single agent TMZ [35]. 

Of particular interest among DNA-damaging agents, poly-ADP-ribose polymerase (PARP) inhibitors are a class of drugs with a potential synergistic effect with checkpoint inhibitors. 

PARP inhibitors are active against cancer cells harboring DDR (DNA damage response) alterations, whose efficacy was initially recognized in breast and ovarian cancers, and currently extended to prostate and pancreatic cancer [107]. In CRC, the role of DDR alterations is still widely unknown and only few data about their clinical impact are available [108].

PARP-based therapies work through the inhibition of single-strand DNA repair and lead to the accumulation of DNA damages, promoting neoantigen release, increasing tumor mutational burden, and enhancing PD-L1 expression.

The preliminary results evaluating the efficacy of PARP inhibitors and immunotherapy are promising in various solid tumors and this strategy is under investigation also in CRC. The DAPPER trial is a phase II basket study of durvalumab with olaparib (PARP inhibitor) or cediranib (VEGFR inhibitor) in refractory pMMR mCRC, advanced pancreatic adenocarcinoma or leiomyosarcoma (NCT03851614) [109]. Consistently with the limited amount of data in this field, the objective of this study is to evaluate the changes in genomic and immune biomarkers in tumor, peripheral blood and stool samples, in addition to changes in radiomic profiles.

## 5. Anti-TAMs + Immune Checkpoint Inhibitors

In advanced cancers immunosuppressive effects mediated by tumor-associated macrophages (TAMs) could be a possible reason for treatment failure with checkpoint inhibitors [107].

Macrophages recruited into TME through inflammatory signals secreted by neoplastic cells can differentiate in TAMs, a key component of the TME with a dominant role in the balance between proliferation and inhibition of tumor growth [110,111]. 

TAMs are educated by different microenvironmental molecules, and can be polarized in multiple phenotypes with a range of functions. The two opposite extremes are the antitumor M1 and the protumor M2 categories [112,113,114]. 

Although macrophages are able to kill tumor cells, some phenotypes of TAMs can also promote angiogenesis by secreting VEGF and suppress immune responses expressing immunosuppressive ligands (PD-L1 and B7) and producing cytokines (IL-10 and TGFβ) that inhibit the development of an effective immune response against cancer [112,113,114]. 

Considering the crucial role of TAMs as suppressive component, the concomitant use of TAM-focused therapeutic inhibitors might be a way to improve immunotherapy effect in pMMR mCRC [115,116].

With this regard, regorafenib is a multitarget tyrosine kinase inhibitor currently approved for the treatment of mCRC patients who have already been treated with, or who cannot be given, other available treatments including fluoropyrimidine, oxaliplatin, irinotecan, anti-VEGF agents and anti-EGFR antibodies (only if RAS wild-type) [78,79]. Regorafenib would be able to generate a more inflamed TME, directly depleting TAMs or reprogramming their phenotypes through multiple mechanisms of action [117,118]. Firstly, by inducing lower vascular density, regorafenib reduces vessels permeability and TME infiltration by TAMs [117]. Secondly, through the inhibition of colony-stimulating factor 1 receptor (CSF1-R), regorafenib reduces the proliferation of TAMs and reprograms TAMs into a subpopulation with antitumor functions [118]. Moreover, it induces attenuation of the IFN-γ-induced PDL-1 expression and the concentration of other immune inhibitor cytokines with consequent re-modulation of TAMs subpopulations [119].

Preliminary observations suggest the existence of a synergism in terms of immunomodulatory activity when regorafenib is combined with immune-checkpoint inhibitors agents in CRC cells [120].

These findings support the concept of overcoming anti-PD-1-resistance using regorafenib in pMMR mCRC.

REGONIVO (EPOC1603) is a phase Ib trial for metastatic gastric and CRC patients who received regorafenib plus nivolumab in a dose-finding part to estimate the recommended dose, followed by expansion cohorts. The colon cohort included 24 chemorefractory mCRC patients with pMMR tumors and one with dMMR mCRC [121]. 

The combination of regorafenib and nivolumab showed an encouraging response rate of 33% in the pMMR cohort with durable responses. Median PFS and OS were 7.9 months and not reached, respectively.

Among mCRC patients with liver metastasis, only two out of 13 experienced objective response (ORR 15%) with regorafenib plus nivolumab. In contrast, patients with lung metastasis achieved better outcomes with an ORR of 50% (8 out of 16). An exploratory analysis demonstrated no relationship between PD-L1 or TMB and efficacy outcomes [121]. 

With regard to safety, during the regorafenib dose-escalation part, the dose of 160 mg was associated with 3 dose-limiting toxicities and 80 mg daily of regorafenib was identified as the optimal dose for the combination with nivolumab [121]. 

These impressive results were not followed by expected confirmations. No responses were detected in a cohort of pMMR mCRC treated with compassionate administration of nivolumab or pembrolizumab in combination with regorafenib in a single United States (U.S.) center. Among five patients (31%) who experienced stable disease as best response, four had no liver metastases [28]. In line with REGONIVO, this finding could suggest that patients with liver metastases are characterized by a lower antitumor immune response and may be less likely to benefit from checkpoint inhibition [121].

A small population of 28 chemo refractory pMMR mCRC patients received the same regimen in a U.S. phase I/Ib trial, including 12 patients in the phase I cohort (according to dose escalation of 80 or 120 mg or 160 mg of regorafenib) and 16 patients in the expanded cohort [122].

Regorafenib (80 mg) has been proven the recommended doses to add to nivolumab in the expanded cohort. Although toxicity was similar to the Japanese study REGONIVO, very modest efficacy was reported with no objective responses [122]. Intrinsic genetic differences between the Asian and the Caucasian populations have been suggested as an explanation for these heterogeneous results, though in the absence of a clear functional hypothesis [29]. 

In order to further explore the activity of this combination, a single-arm phase II trial (NCT04126733) is currently ongoing [123].

More recently, results of the phase II REGOMUNE trial assessing the activity of avelumab plus regorafenib (at the dose of 160 mg) in refractory pMMR mCRC patients were presented [36].

Among 48 enrolled patients no objective responses were detected while 42.5% and 57.3% experienced progressive disease and stable disease as best response, respectively. In this study, PFS and OS results were also quite modest: 3.6 and 10.8 months, respectively [36]. 

Safety data were consistent across these studies highlighting that regorafenib at the dose of 160 mg was associated with a high incidence of severe adverse events [28,36,121,122]. This is not surprising in the light of the escalating flexible dosing approach currently recommended and widely adopted in the daily clinical practice. Based on findings of the REDOS and REARRANGE trials, escalating strategies are considered feasible alternatives to the standard dose of 160 mg/day to reduce the toxicity burden while not impairing clinical outcomes [30,124]. 

However, inconsistent results about the combination of regorafenib and checkpoint inhibitors in different trials clearly indicate that much more biological knowledge is needed to disclose the reasons of effective vs. ineffective TME modulation.

An ancillary planned analysis in REGOMUNE trial showed that higher TAMs infiltration detected on tumor samples at baseline was significantly associated with shorter median PFS [36].

Additional analyses with larger sample size will be essential to clarify the role of TME composition as a biomarker of treatment outcomes with this combination.

Similarly to regorafenib, preclinical data demonstrated that lenvatinib, an oral multikinase inhibitor, reduced and/or reprogrammed TAMs. In the phase II LEMON trial, lenvatinib showed a promising DCR with an acceptable safety profile in refractory mCRC patients [125].

Consistently with results from other solid tumors, lenvatinib plus PD-1/PD-L1 inhibitors seem a well-tolerated regimen with a potent antitumor activity.

Based on the hypothesis that regorafenib or lenvatinib may work in concert with anti PD-1/PD-L1, phase I/II trials of regorafenib or lenvatinib given together with pembrolizumab in pretreated mCRC patients are ongoing (NCT03657641 and NCT03797326) [37,126].

## 6. Radiotherapy + Immune Checkpoint Inhibitors

The synergy between immune checkpoint inhibitors and radiotherapy (RT) is well-documented in literature and a daily reality in the clinical practice of unresectable non-small-cell lung cancer (NSCLC) [38,127,128,129]. 

By damaging DNA, RT induces tumor-cell death promoting antigen presentation, CTLs recruitment and activation and up-regulation of inflammatory cytokines [130,131,132,133,134,135,136,137]. 

In preclinical models, the immunologic impact of different RT fractionation schedules remains controversial and deserves further investigation, given the heterogeneous radiation protocols used in the clinical practice [138,139,140,141]. Here, we discuss both locally-advanced rectal cancer (LARC) setting, which is the routine field of application of RT in CRC, and the metastatic disease, in which RT has recently turned into an appealing approach to reinvigorate anti-tumor immunity, and consequently the efficacy of immunologic agents [142,143,144]. 

### 6.1. Locally Advanced Rectal Cancer

The combination of fluoropyrimidine and RT in the neoadjuvant setting is the current standard of care in LARC and exploits the synergy between radiation and systemic treatment to prevent local and distant relapse [143,144]. Nonetheless, distant recurrence remains a frequent event (25–30% of resected patients) and novel approaches are needed to improve patients’ survival outcome [145,146]. Therefore, together with the growing evidence of efficacy of pre-surgical chemotherapy intensification, the combination of chemo-radiotherapy (CRT) and immune checkpoint inhibitors is considered as a promising strategy to promote the response of the primary tumor, while possibly boosting an immunological memory to further reduce the risk of local and distant metastases in pMMR LARC [38,147,148]. 

Drawing from these evidences, several studies are currently addressing this issue in this critical clinical landscape. The phase II AVANA trial explores the combination of neoadjuvant capecitabine-based CRT + avelumab to assess the added value of administering an immune checkpoint inhibitor to the current approved standard of care strategy for the management of LARC (NCT03854799). The primary objective of this trial is the rate of complete pathologic response (pCR) [149].

Neoadjuvant intensified chemotherapy plus RT ± concomitant capecitabine associated with immune checkpoint inhibitors is being assessed in two phase II clinical trials (NCT04411537, NCT03921684) [39,40]. 

Other ongoing approaches are assessing in pMMR LARC patients chemo-free neoadjuvant protocols, as the PEMREC trial (RT + Pembrolizumab) (NCT04109755) and TARZAN trial (RT + Atezolizumab + Bevacizumab) (NCT04017455) [41,42]. 

### 6.2. Metastatic Disease

The immune mediated response to RT of tumor cells located out of the field of irradiation (abscopal effect) is a remote concept in oncology, though still of limited application in the routine clinical practice of metastatic disease [142]. Indeed, only 46 case reports of abscopal effect from RT have been documented in 45 years [43]. However, the immune checkpoint era has established a renewed interest in the abscopal effect, after the discovery that anti-PD1/PD-L1 agents can synergize with locoregional radiation to boost a regression in off-target lesions [150,151,152]. 

In the clinical setting, the combination of RT and immunotherapy is currently being explored in two potential fields of application: the widespread metastatic setting, where RT is expected to synergize with response to immunotherapy in sites of metastasis out of the field of irradiation, as a consequence of the aforementioned promotion of neoantigen release and T cell activation; or the oligometastatic disease, in which patients undergo locoregional RT with a curative intent and immunotherapy agents may serve to boost both a response within the field of radiation, and the immunological memory in order to prevent local and distant relapse [127,142]. 

Although available efficacy data of RT associated with checkpoint inhibitors in mCRC are limited, several studies suggest a generally tolerable toxicity profile associated with this approach. 

Dual blockade of ipilimumab and nivolumab with RT (8 Gy in 3 fractions to a single metastatic lesion) demonstrated feasibility and promising activity in a phase II study that included 40 patients with refractory pMMR mCRC [153].

Several phase II clinical trials are currently assessing the activity of immune checkpoint inhibitors added to different strategies of locoregional radiation (standard RT, stereotactic body radiotherapy [SBRT], radiofrequency ablation [RFA]) in the same setting [31,44,45]. Interestingly, one of these studies encompasses two different regiments of RT fractionation, each one tested with the combination of durvalumab + tremelimumab (NCT02888743) [46]. 

Another phase II study is investigating the addition of regorafenib to the combination of RT + nivolumab (NCT04030260) [47]. 

With regard to the oligometastatic setting, one phase II and two small phase I clinical trials are exploring the synergy of immune checkpoint inhibition + SBRT, in one case in combination with a conversion chemotherapy with XELOX + apatinib, a VEGFR-2 inhibitor (NCT03927898, NCT02837263, NCT04202978) [48,49,50,51]. In liver-predominant pMMR mCRC patients, one phase 1/2 trial is evaluating the feasibility and safety of yttrium-90 radioembolization in combination with a fixed dose of durvalumab (750 mg) (NCT04108481) [52]. 7. Target Therapy + Immune Checkpoint Inhibitors.

### 6.3. MAPK Signaling

The extracellular-signal regulated kinases (ERK) have been extensively explored in the pathogenesis of CRC as the downstream effectors of multiple receptor tyrosine kinase, including the family of Mitogen-Activated Protein Kinases (MAPK) [52,154]. Activation of the MAPK/ERK cascade is downstream multiple growth factor receptors including EGFR, target of anti-EGFR monoclonal antibodies cetuximab and panitumumab currently available for RAS wild type mCRC [155,156,157,158]. 

A growing amount of evidence shows that mCRC harboring an activating mutation in the RAS/BRAF/MEK/ERK pathway are associated with an immunosuppressive phenotype which is determined by an intriguing interplay between MAPK signaling and mechanisms of immune-evasion, including but not limited to the expression of immune checkpoints [159,160,161,162,163,164,165,166]. 

Drawing from these findings, there is an appealing rationale to investigate the synergic effect of immunotherapy agents and selective inhibitors of the RAS/BRAF/MEK/ERK pathway in pMMR CRC. 

#### 6.3.1. KRAS Targeting

Activating mutations of RAS genes have been found to shape an immunosuppressive phenotype in CRC [160]. Notably, KRAS mutated CRC are characterized by limited cytotoxic cells (CTLs, NK cells) infiltration and blunted IFN-γ signaling, a key regulator of PD-L1 expression [160]. 

Indeed, in the phase III KEYNOTE 177 study, OS subgroup analysis led to hypothesize less benefit from pembrolizumab in RAS mutated dMMR mCRC than standard of care, suggesting that the immunosuppressive microenvironment due to RAS mutations may limit the efficacy of immunotherapy also in the favorable subset of dMMR tumors [6]. 

Mechanistically, this can be explained by the RAS-driven repression of interferon regulatory factor 2 (IRF2), resulting in subsequent expression of CXCL3, a chemokine that attracts MDSCs to the TME [158]. Owing to its molecular architecture, RAS oncoprotein has been dismissed as “undruggable” for long and even targeting its downstream effectors has not achieved satisfactory clinical efficacy in CRC [167]. 

Nevertheless, the discovery of KRAS inhibitors such as MRTX849 and AMG510 has recently questioned this dogma, at least in the subgroup of CRC harboring a KRAS G12C mutation [162,168]. 

Preliminary data of a phase II ongoing trial show promising DCR (76%), but markedly lower ORR (7%) in pretreated KRAS G12C mutated patients treated with the KRAS G12C inhibitor sotorasib (AMG510) [53].

In the dose-expansion cohort, including patients receiving 960 mg once-daily, the ORR was 12% and the DCR was 80%. Median PFS was 4.2 months and OS was unreached after a median follow-up of almost 8 months [53]. The upstream activation of EGFR, similarly to what occurs in BRAF V600E mutated mCRC patients treated with anti-BRAF monotherapy, may explain the low ORR observed in this study, as recently shown by Amodio et al. in a pre-clinical model of CRC [169].

Remarkably, in preclinical models AMG510 shows tumor killing ability as monotherapy or when combined with other therapies and it results in a pro-inflammatory TME, even in presence of KRAS G13D mutations, suggesting a rationale for combinations of KRAS-inhibitors and immunotherapeutic agents, also in the case of heterogeneous KRAS G12C abundance [162]. 

Based on this evidence, AMG510 in combination with anti-PD-1/PD-L1 is being investigated in patients with KRAS G12C mutated mCRC (NCT03600883) [54]. According to pre-clinical findings by Amodio et al., incorporating also an anti-EGFR in this combination may potentiate the clinical efficacy of KRAS G12C and PD-1/PD-L1 inhibition [169]. A futuristic direction to emphasize efficacy of immunotherapy approaches in KRAS mutated pMMR mCRC might be the combination of checkpoint inhibitors and/or triggers of IRF2 axis (i.e., inhibitors of the CXCL3 receptor CXCR2).

#### 6.3.2. BRAF Inhibition

A hallmark of poor prognosis in mCRC patients is BRAF V600E mutation (8–10%), that is associated with dMMR of sporadic origin in around 30% of cases [170,171,172,173]. This is explained by the widespread methylation of CpG islands and the consequent silencing of MLH1 promoter, resulting in genome hypermutation that seems at least in part explained by the downstream recruitment of a corepressor complex binding to MLH1 promoter [174,175,176]. 

Data from trials evaluating immunotherapies in dMMR mCRC patients reported similar results independently of BRAF mutational status, thus strengthening the role of these agents in the therapeutic route of patients bearing BRAF V600E mutated mCRC [5,6,177]. 

On the other side, inhibiting BRAF in BRAF V600E in pMMR CRC could help mirroring the immune-inflamed CRC phenotype of dMMR tumors. Since BRAF inhibition alone has proved ineffective in mCRC, the co-administration of anti-EGFR to prevent the feedback reactivation of EGFR and of the downstream immune-suppressive MAPK cascade, appears as a biologically sound strategy [178,179]. 

In fact, the BEACON trial was designed to compare the triplet encorafenib (BRAF inhibitor), binimetinib (MEK inhibitor) and cetuximab vs. a standard of care in pretreated BRAF mutated mCRC patients. 665 patients were assigned to the triplet targeted therapy with binimetinib, encorafenib, and cetuximab, the doublet targeted therapy with encorafenib and cetuximab, or the control treatment cetuximab and irinotecan +/− 5FU [179]. This given, the identical OS advantage observed in the triplet and doublet arms [median OS: 9.3 months, 95% Confidence interval (CI) (8.1–10.8) and 9.3 months, 95% CI (8.0–11.3)] vs. the control arm [5.9 months, 95% CI (5.1–7.1)] has questioned the utility of simultaneous MEK inhibition [179]. Based on these results, the combination of encorafenib and cetuximab is now identified as a standard therapeutic option in BRAF mutated mCRC patients who had had disease progression after one or two previous treatment regimens [180]. 

No significant interaction between treatment and microsatellite status was evident, but the low percentage of dMMR patients recruited (8%) suggests that this subgroup of patients was underrepresented and the study not sufficiently powered to detect a statistically significant treatment effect of BRAF + EGFR ± MEK inhibition according to the microsatellite status [179]. 

Recently, preclinical data showed that BRAF and EGFR blockade synergizes with immune checkpoint inhibitors in in vitro models of BRAF mutated CRC in presence of simultaneous pharmacological inhibition of autophagy [181,182,183]. This intriguing synergy may be explained by the fact that tumors with upregulation of the MAPK pathway exploits autophagy-dependent MHC-I degradation as a critical mechanism to evade immune surveillance [184]. Therefore, cooperative BRAF and autophagy inhibition may preserve MHC-I exposure and antigen expression on tumor cell surface, thus resulting in synergic improved activity of immune checkpoint inhibitors. 

Drawing from this evidence, one phase I/II and one phase II clinical trials are currently investigating the activity and efficacy of BRAF + MEK + PD-1 inhibitors in stage III and IV CRC patients harboring an activating BRAF mutation (NCT04044430 and NCT03668431) [55,56]. 

Preliminary results of this strategy showed that the combination is well tolerated with a favorable response rate (ORR 33%) in BRAF V600E mutated mCRC patients [185].

Future studies may incorporate a pharmacological inhibitor of autophagy to challenge the pre-clinical evidence of MAPK and autophagy inhibition synergism with immune checkpoint inhibitors also in a clinical setting. 

### 6.4. PIK3CA/AKT/mTOR Targeting

Fully integrated with the MAPK signaling, the PIK3/AKT/mTOR pathway drives cell survival, proliferation, angiogenesis and migration downstream EGFR and several other tyrosine kinase receptors [186,187,188]. PIK3 acts as a crucial node in this molecular network, establishing a crosstalk between RAS and AKT/mTOR signaling, and harbors an activating mutation in the 7–32% of mCRC [188]. Nonetheless, the prognostic and predictive role of PIK3 in mCRC has not been fully characterized yet, also because of the frequent co-occurrence of this alteration with RAS mutations [189,190,191,192]. 

In addition, clinical testing of PIK3 inhibitors in solid tumors has been hampered by major metabolic side effects, especially hyperglycemia, and the possible undesired blockade of white blood cell proliferation, under the control of PIK3/AKT/mTOR pathway [193,194]. However, a renewed interest in PIK3 and anti-PD1/PD-L1 combinations has been established by recent findings that PIK3γ and PIK3δ isoforms reprogram TAMs towards an immune-tolerant phenotype, at the same time downregulating tumor-infiltrating myeloid cells [195,196,197].

Consistently with these findings, one phase I/II clinical trial is currently investigating the combination of Nivolumab + Copanlisib, a PIK3 inhibitor, in pMMR mCRC patients (NCT03711058) [57]

## 7. Conclusions

The success of checkpoint inhibitors in determining durable responses and prolonging survival in dMMR mCRC patients heralded a new dawn in the treatment of this subgroup. However, about 95% of mCRC patients are excluded from this excellent therapeutic option. 

In order to make pMMR mCRC responsive to immunotherapy, efforts are focused on combination strategies aiming at turning “immune tolerant” tumors into “immune competent” ones. Some failures have been reported up today but several studies that lay foundation on strong biologic and clinical bases are currently ongoing. Further insight into the mechanisms of immunotherapy resistance and the heterogeneous spectrum of mCRC is needed in order to improve pharmacological strategies to overcome primary resistance to immunotherapy. To this end, substantial help is awaited from translational research with the aim of building truly personalized individual approaches.

## Figures and Tables

**Table 1:** Selection of completed trials investigating the use of immune checkpoint inhibitors for proficient mismatch repair (pMMR) or non-specified microsatellite status (MSI n.s.) colorectal cancer.

Study Nameand/or NTC	Phase	Study Population	Agent(s)	End-Points *	Results
**Anti-VEGF and Chemotherapy + Immune Checkpoint Inhibitors**					
NCT0237335672 [21]	II	1st line mCRC MSI n.s.	FOLFOX + Pembrolizumab	1: PFS 2: ORR, DCR	mPFS not reached ORR: 53%, DCR: 100%
MODUL cohort 2 NCT02291289 [22]	II	1st line mCRC BRAF wt, MSI n.s.	FOLFOX + BV followed by FP + BV vs. FP + BV + Atezolizumab	1: PFS 2: OS	PFS: 7.4 mos vs. 7.2 mos, HR 0.96, *p* = 0.727 OS: 51%, HR = 0.86, *p* = 0.28
BACCI NCT02873195 [23]	II	Pretreated mCRC MSI n.s.	Capecitabine + BV + Atezolizumab vs. Capecitabine + BV	1: PFS 2: 12 mo OS	mPFS: 4.4 mos vs. 3.3 mos. HR 0.72, *p* = 0.051 12 mo OS: 43% vs. 52%, HR 0.94, *p* = 0.4.
NCT01633970 [24]	Ib	1st line mCRC MSI n.s.	FOLFOX + BV + Atezolizumab	1: Safety 2: PFS, OS	mPFS: 14.1 mos OR: 52%
ATEZOTRIBE NCT03721653 [25]	II	1st line mCRC MSI n.s.	FOLFOXIRI + BV + Atezolizumab vs. FOLFOXIRI + BV	1: PFS 2: ORR, irORR, R0 resection rate	No results posted
**Anti-EGFR ± Chemotherapy + Immune Checkpoint Inhibitors**					
AVETUX NCT03174405 [26]	II	1st line mCRC RAS/BRAF wt MSI n.s.	FOLFOX + Cetuximab + Avelumab	1: 12 mos PFS 2: PFS, ORR	12 mo PFS: 40% mPFS: 11.1 mos ORR: 79.5%
CAVE Eudract 2017-004392-32 [27]	II	Pretreated mCRC RAS wt, MSI n.s.	Cetuximab + Avelumab	1: OS 2: ORR, PFS, Safety	No results posted
**Anti-TAMs + Immune Checkpoint Inhibitors**					
REGONIVO NCT03406871 [28]	Ib	Preatreatd mCRC MSI n.s.	Regorafenib + Nivolumab	1: Safety 2: ORR, PFS, OS	ORR: 36% mPFS: 5.6 mos mOS: not reached
NCT03712943 [29]	I/Ib	Preatreatd mCRC pMMR	Regorafenib + Nivolumab	1: Safety 2: ORR, PFS, OS	ORR: 5% mPFS: 4.3 mos mOS: 11 mos
REGOMUNE NCT03475953 [30]	II	Pretreated mCRC pMMR	Regorafenib + Avelumab	1: ORR 2: PFS, OS	ORR: 0% mPFS: 3.6 mos mOS: 10.8 mos
**Radiotherapy + Immune Checkpoint Inhibitors**					
NCT03104439 [31]	II	Preatreatd mCRC pMMR	SBRT 8 Gy + Nivolumab + Ipilimumab	1: DCR 2: ORR	DCR: 17.5% ORR: 7.5%

* Primary end-points (1) and authors’ selected secondary end-points (2) are listed. Abbreviations: BV: Bevacizumab; DCR: Disease Control Rate; EGFR: Epidermal Growth Factor Receptor; FOLFOX: 5-FU + Folinic Acid + Oxaliplatin; FP: Fluoropyrimidine; HR: Hazard Ration; irORR: immune-related Overall Response Rate; mCRC: Metastatic Colorectal Cancer; mos: months; ORR: Overall Response Rate; OS: Overall Survival; PFS: Progression Free Survival; SBRT: Stereotactic Body Radiation Therapy; TAMs: Tumor-Associated Macrophages; VEGF: Vascular Endothelial Growth Factor.

**Table 2:** Selection of ongoing trials investigating the use of immune checkpoint inhibitors for proficient mismatch repair (pMMR) or non-specified microsatellite status (MSI n.s.) colorectal cancer.

Study Name and/or NTC	Phase	Study Population	Agent(s)	Primary End-Point(s)
**Chemotherapy + Anti-Angiogenetics + Immune Checkpoint Inhibitors**				
NIVACOR NCT04072198 [32]	II	1st line mCRC RAS/BRAF mt, MSI n.s.	FOLFOXIRI + BV + Nivolumab	ORR
**Anti-EGFR ± Chemotherapy + Immune Checkpoint Inhibitors**				
AVETRIC Eudract 2019-001501-24 [33]	II	1st line mCRC RAS wt, MSI n.s.	FOLFOXIRI + Cetuximab + Avelumab	PFS
**Temozolomide + Immune Checkpoint Inhibitors**				
ARETHUSA NCT03519412 [34]	II	Pretreated mCRC RAS mt, MGMT-neg pMMR (Cohort P)	TMZ until progression followed by Pembrolizumab if TMB > 20 Muts/Mb after TMZ administration	ORR
MAYA NCT03832621 [35]	II	Pretreated mCRC MGMT-neg, pMMR	TMZ for 2 cycles followed by TMZ + Nivolumab + Ipilimumab if SD/PR/CR to TMZ monotherapy	8 mo PFS
**Anti-TAMs +** **Immune Checkpoint Inhibitors**				
NCT04126733 [36]	II	Pretreated mCRC BRAF wt pMMR	Regorafenib + Nivolumab	ORR
NCT03657641 [37]	I/II	Pretreated mCRC MSI n.s.	Regorafenib + Pembrolizumab	Safety, PFS, OS
NCT03797326 [38]	II	Pretreated mCRC pMMR	Lenvatinib + Pembrolizumab	ORR, Safety
**Radiotherapy + Immune Checkpoint Inhibitors**				
AVANA NCT03854799 [39]	II	LARC MSI n.s.	Pre-op capecitabine + RT + Avelumab → Surgery	pCR
NCT04411537 [40]	II	LARC pMMR	Pre-op Nivolumab → CAPIRI + RT → Nivolumab → Surgery → post-op XELOX	pCR
NCT03921684 [41]	II	LARC MSI n.s.	Pre-op capecitabine + RT → FOLFOX + Nivolumab → Surgery	Safety, pCR
PEMREC NCT04109755 [42]	II	LARC pMMR	Pre-op Short-Course RT + Pembrolizumab → Surgery	TRG
TARZAN NCT04017455 [43]	II	LARC MSI n.s.	Pre-op bevacizumab + atezolizumab → Surgery	cCRR and near-cCRR
NCT02437071 [44]	II	Pretreated mCRC MSI n.s.	Pembrolizumab + RT or RFA	ORR out of field of radiation
NCT03122509 [45]	II	Pretreated mCRC pMMR	Durvalumab + Tremelimumab + RT or RFA	ORR out of field of radiation
SABR-PDL1 NCT02992912 [46]	II	Pretreated mCRC MSI n.s.	Atezolizumab + SBRT (45 Gy)	PFS
NCT02888743 [47]	II	Pretreated mCRC pMMR	Durvalumab + Tremelimumab + High dose RT (Arm B) or Low dose RT (Arm C) vs. Durvalumab + Tremelimumab (Arm A)	ORR out of field of RT of Arm B and ARM C vs. ORR in Arm A
NCT04030260 [48]	II	Pretreated mCRC pMMR	Regorafenib + Nivolumab + RT ± irinotecan	mPFS
NCT03927898 [49]	II	Oligometastatic CRC with resected primary, achieving SD or PR to first-line therapy, and with all lesions amenable to SBRT MSI n.s.	SBRT + Toripalimab	12 mo PFS
NCT02837263 [50]	Ib	Pretreated CRLM, candidate for SBRT to at least one intrahepatic lesion and for surgery with potential curative intent. pMMR	SBRT + Pembrolizumab	12 mo recurrence rate
NCT04202978 [51]	I/II	Neoadjuvant CRLM. MSI n.s.	CAPOX + Apatinib + Camrelizumab → RFA → Surgery	R0 resection rate
iRE-C NCT04108481 [52]	I/II	Liver-predominant mCRC	Yttrium-90 RadioEmbolization + Durvalumab	Safety
**Target Therapy + Immune Checkpoint Inhibitors**				
CodeBreak 100 NCT03600883 [53]	I/II	Pretreated mCRC KRAS G12C mt MSI n.s.	AMG510 (Sotorasib) + Pembrolizumab	Safety
CodeBreak 101 NCT04185883 [54]	Ib	Pretreated mCRC KRAS G12C mtMSI n.s.	AMG510 (Sotorasib) + anti-PD1	Safety
NCT04044430 [55]	I/II	Pretreated or PD within 6 mos of post-op CT mCRC BRAF V600E mt pMMR	Encorafenib + Binimetinib + Nivolumab	Safety, ORR
NCT03668431 [56]	II	Pretreated mCRC BRAF V600E mt MSI n.s.	Dabrafenib + Trametinib + Spartalizumab	Safety, ORR
NCT03711058 [57]	I/II	Pretreated mCRC pMMR	Copanlisib + Nivolumab	Safety, ORR

Abbreviations: BV: Bevacizumab; CAPOX: Capecitabine + Oxaliplatin; cCRR: clinical Complete Response Rate; CR: Complete Response; CRLM: Colorectal Liver Metastases; CRC: Colorectal Cancer; CT: Chemotherapy; EGFR: Epidermal Growth Factor Receptor; FOLFOX: 5-FU + Folinic Acid + Oxaliplatin; FOLFOXIRI: 5-FU + Folinic Acid + Oxaliplatin + Irinotecan; LARC: Locally Advanced Rectal Cancer; mCRC: Metastatic Colorectal Cancer; mos: months; MGMT: O6-Methylguanine-DNA Methyltransferase; Muts/Mb: Mutations per Megabase; ORR: Overall Response Rate; OS: Overall Survival; PFS: Progression Free Survival; PR: Partial Response; pre-op: pre-operatory; post-op: post-operatory; RFA: Radiofrequency Ablation; RT: radiotherapy; SBRT: Stereotactic Body Radiation Therapy; SD: Stable Disease; TAMs: Tumor-Associated Macrophages; TMB: Tumor Mutational Burden; TMZ: Temozolomide; TRG: Tumor Regression Rate; VEGF: Vascular Endothelial Growth Factor.
